# An evaluation of a checklist in musculoskeletal radiographic image interpretation when using artificial intelligence

**DOI:** 10.1002/jmrs.850

**Published:** 2024-12-20

**Authors:** Laura McLaughlin, Sonyia L McFadden, Angelina T Villikudathil, Jonathan McConnell, Ciara Hughes, Raymond Bond, Clare Rainey

**Affiliations:** ^1^ School of Health Sciences, Faculty of Life and Health Sciences Ulster University Belfast Northern Ireland UK; ^2^ Diagnostic Radiography, School of Health and Society University of Salford Manchester UK; ^3^ School of Computing, Faculty of Computing, Engineering and the Built Environment Ulster University Belfast Northern Ireland UK

**Keywords:** artificial intelligence, checklist, image interpretation, musculoskeletal

## Abstract

**Introduction:**

Artificial intelligence (AI) is being used increasingly in image interpretation tasks. Human reliance on technology and bias can cause decision errors. A checklist, used with the AI to mitigate against such biases, may optimise the use of AI technologies and promote good decision hygiene. A checklist to aid radiographic image interpretation for radiographers using AI for image interpretation was formed. This study investigates the effect of a checklist for musculoskeletal (MSK) radiographic image assessment when using AI interpretive assistance.

**Methods:**

Radiographers were asked to interpret five MSK examinations with AI feedback. They were then provided with the checklist and asked to reinterpret the same five examinations with the AI feedback (*n* = 140 interpretations). During the interpretation sessions, participants were asked to provide a diagnosis and a confidence level on the diagnosis provided. Participants were then asked to complete a questionnaire to gain feedback on the use of the checklist.

**Results:**

Fourteen radiographers were recruited. Nine participants found the checklist alongside the AI most useful and five participants found the AI element to be most useful on its own. Five participants found the AI feedback to be useful as it helped to critique the radiographic image interpretation more closely and rethink their own initial diagnosis.

**Conclusion:**

The checklist for use with AI in MSK image interpretation contained useful elements to the user, but further developments can be made to enhance its use in clinical practice.

## Graphical abstract

A checklist to aid radiographic image interpretation for radiographers using AI for image interpretation was formed. Radiographers were asked to interpret five musculoskeletal (MSK) examinations with AI feedback before and after the use of the checklist. The checklist for use with AI in MSK image interpretation contained useful elements to the user but further developments can be made to enhance its use in clinical practice.

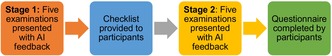

## Introduction

### Increasing use of AI in diagnostic radiography

AI as a human adjunct in diagnosing pathology on radiographic images began in the 1960s with the development of a system of conversion of an image to numerical data.^1^ Differing methods of analysis of medical, and other, images have been developed. Deep learning (DL) methods using convolutional neural networks (CNN) is the most recent and seemingly most promising form of AI for detection of disease on radiographic images. Accuracies of DL algorithms of up to 97% for detection of specified pathology,^2^ have been reported. Applications of DL algorithms on skeletal radiographs have been less extensively investigated, but a small number of studies suggest that AI also has a place in detection of osteoporosis, bone age and fracture detection.^3^ These developments have been the focus of much media and professional attention, and more recently, AI has been targeted as an area of focus for modernising and future‐proofing the National Health Service in the United Kingdom (UK).^4^ AI has been proposed as a solution in automated diagnosis of pathology on medical images, for example breast, skeletal and chest imaging.^4^, ^5^, ^6^ This is particularly important in a health service coping with the current waiting lists as a consequence of the COVID‐19 global pandemic and where resources are limited.

Plain radiographic studies account for a significant proportion of the overall imaging demand in the UK. Therefore, potential cost and time efficiency for interpretation of examinations may have the most significant impact in this area, allowing a reallocation of resources to areas of greater need in today's NHS landscape.

### Bias and Trust in using AI

Biases are evident in the literature whereby the user questions and changes their decision due to reliance on the information provided by AI.^7^, ^8^, ^9^ Issues may arise where the user is biased towards the AI information, leading to an incorrect decision.^10^ Despite reported accuracies and benefits, clinicians' trust in AI remains a barrier to implementation in the healthcare setting.^11^, ^12^ DL is one form of AI where algorithms use multiple neural layers to analyse and process image data, but with many layers hidden to the user to generate doubt in ultimate system decision making or a so‐called ‘black‐box’. Attempts are currently being made to make this process more transparent using, for example heat or salience maps as visual representations of AI attention.^13^, ^14^

### Methods to reduce bias and decrease variation – Decision hygiene

In the past, radiographers have drawn on various checklists to aid their interpretation of images and to ensure thorough image interrogation is completed. With the additional element of AI in the interpretation process, a checklist may ensure all aspects are considered. A checklist alongside AI use could reinforce the human responsibility and reasoning inputs rather than merely agreeing with AI outputs. The checklist enables a ‘decision hygiene’ element to be added to the interpretation process, promoting the use of conscious reasoning rather than intuition linked to diagnosis.^14^ Checklists have been used previously to aid image interpretation processes and have been considered a promising intervention for the area of diagnosis.^15^, ^16^ The use led to positive effects on participant performance, with a decrease in false positives and increase in true negatives identified.^17^ Checklists should be short to reduce cognitive load on the user and maximise performance.^18^ Contrary to this, checklists have the potential to cause users to question their initial decision or can lead to little or no improvements.^19^ By incorporating reminders in a checklist, which the AI may not have the capacity to consider, the use of AI in image interpretation practice could improve. Decision rationalisation, avoidance of over reliance on the technology and promotion of appropriate trust in AI systems for decision support in radiographic image interpretation becomes an improved norm.

This research is important to reduce variation in the impact of AI amongst radiographers completing Preliminary Clinical Evaluation (PCE) and optimise the use of AI in clinical decision support in image interpretation.

#### Study objective

To test the effect of a checklist for MSK radiographic image assessment when using AI interpretive assistance.

## Materials and Methods

### Recruitment

Participants were recruited between February and April 2022 through contacts in educational institutions, attendance at a European conference and attendance at post‐graduate programs in the UK. An introductory letter and consent form were provided to participants online and written informed consent was obtained prior to participation in this study. The study was provided to participants using an online link, and they completed the entire image interpretation section using a laptop.

### Inclusion criteria

Participants willing to dedicate their time to the study, qualified radiographers, radiographers commonly completing PCE in clinical practice and signed written informed consent from participants.

### Exclusion criteria

Withdrawal of consent or participation in the study.

### Study structure

**Figure 1:**
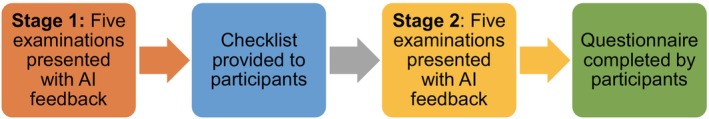
Layout of the study design.

Participants interpreted five MSK examinations with AI assistance and then re‐interpreted the same five examinations, with AI assistance, whilst using the checklist. The MSK images were presented as ‘patient examinations’ (i.e. containing more than one radiographic image) to replicate the image interpretation task in clinical practice. Images were provided with AI feedback in both the initial and follow‐up image interpretations. In the follow‐up image interpretation task, participants had access to the checklist, to determine its impact and inter‐rater reliability. Accuracy in diagnosis and confidence in the diagnosis were evaluated before and after the introduction of the checklist. Participants were not time restricted when completing the study and were therefore able to take a break during the study if required. A questionnaire was provided to participants following the after intervention data collection session. A layout of the study design is presented in Figure 1. The participants were provided with examination forms in the two stages: A checklist form was provided for use during stage two, and a questionnaire was provided electronically online.

### Image content and AI

Musculoskeletal (MSK) images were selected from a previously compiled test bank available for use in research.^20^ The AI used in this study is a CNN, which has been developed and utilised in other research studies for identification of fractures on appendicular skeletal radiographs^20^ The CNN Is based on a ResNet‐152 architecture, pre‐trained on ImageNet and further trained by transfer learning on clinical MSK images from the MURA dataset of 40,561 images.^21^ There is no explicit test set for the MURA data, so a proportion of the validation set was used (783 patients, 1199 studies and 3197 images) as the test set and the rest as the validation set. There was no overlap between any of the sets. The arithmetic mean of the output was calculated and used to determine pathology with a threshold value of 0.5 for pathology. Training continued until no more improvement was found. Optimisation using Adam was used with an initial learning rate of 1 × 10^−4^. Following training and validation using MURA, the model was used to detect pathology on clinical images. A binary saliency map was produced as a form of explainability. This was based on a technique described by Kumar et al., 2018.^22^

**Figure 2:**
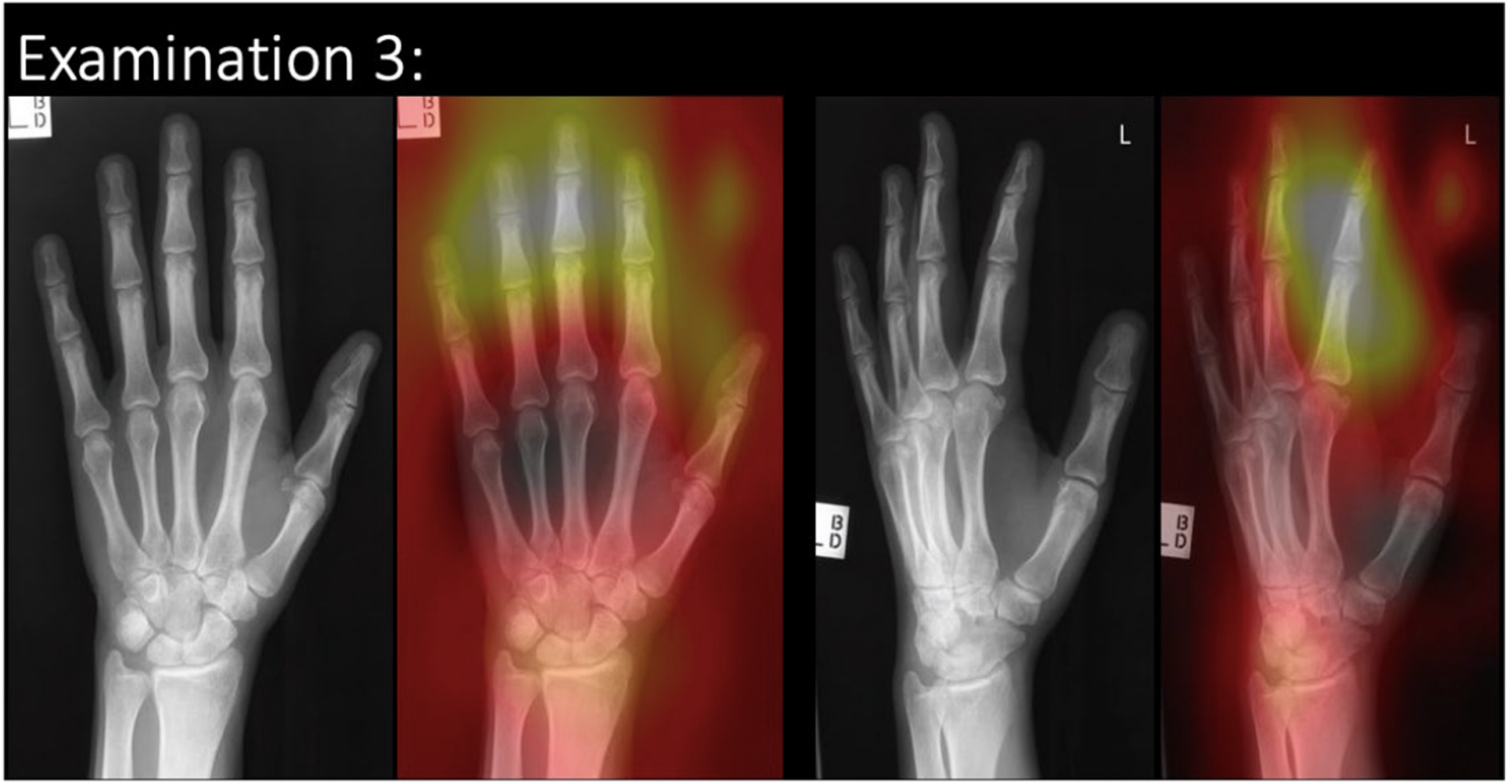
Example of the radiographic image examination shown to the participants.

Agreement with the ground truth on the clinical images was found to be little better than chance, at 57.1% accuracy ((incorrect predictions/total) × 100); however, for this study, only images where the AI determined the correct diagnosis were used. Each examination has a consensus diagnosis from three to five expert clinicians (reporting radiographers or radiologists). This was taken as ground truth diagnosis, as per best practice reported in other studies.^13^, ^20^, ^23^, ^24^ AI feedback was available in Stage 1 and Stage 2 as a heatmap identification of area of AI focus. An example of the radiographic examination image is present in Figure 2. The image/examination bank provided to participants was unchanged before and after access to the checklist to allow the impact of the checklist to be determined. Two of the five examinations contained a pathology. The remaining three examinations did not contain a pathology. Participants were asked to provide a diagnosis and confidence level on the diagnosis for each examination.

### Checklist

**Figure 3:**
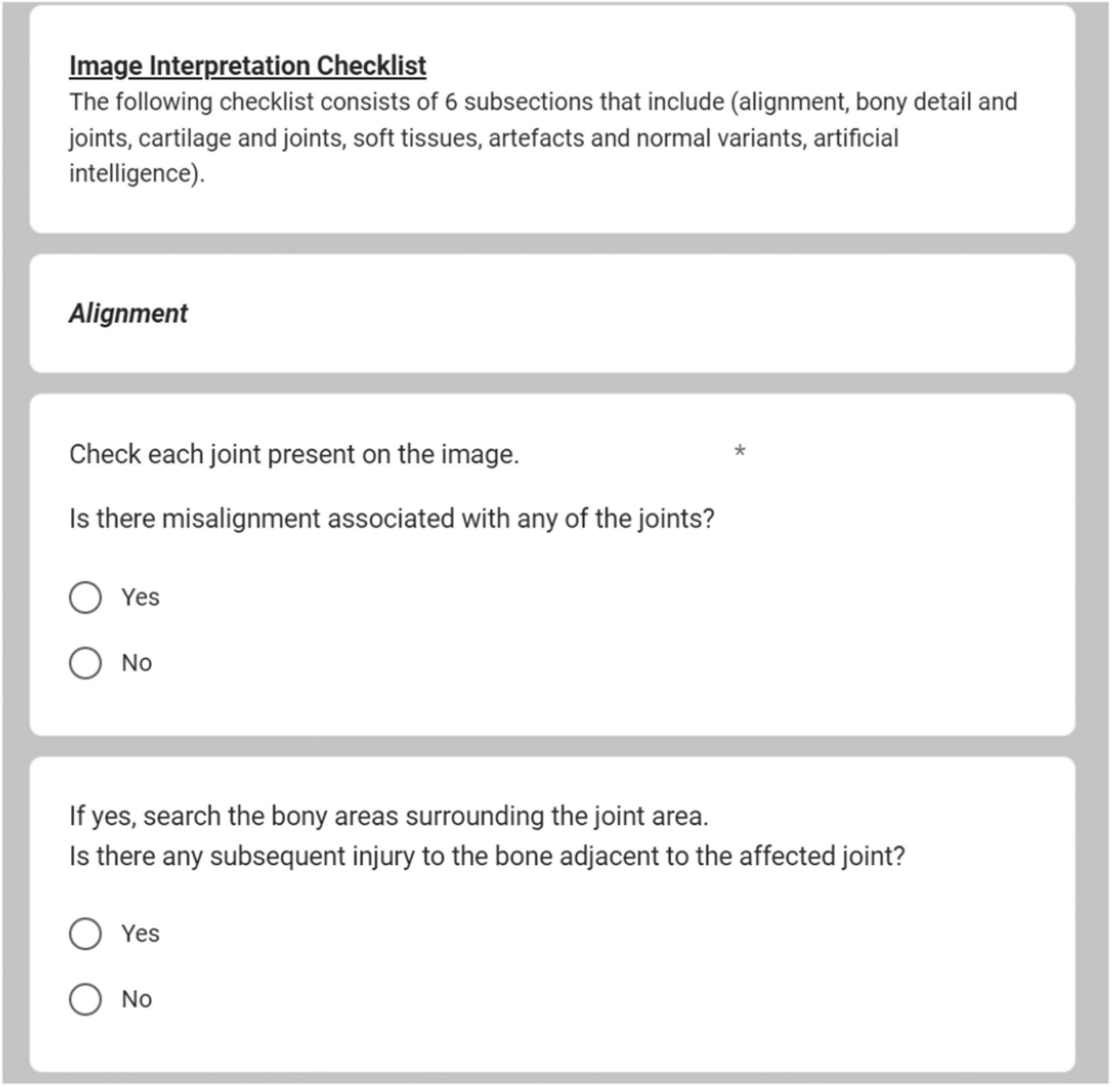
Section of the checklist provided to participants.

A checklist to aid the systematic search of an image for pathology(ies) was formed from expert consensus and followed the principles developed using expert consensus and collaboration as described in chest image interpretation performance of reporting radiographers.^25^ The checklist contained six sections: general considerations, artefacts and normal variants, soft tissues, bony detail and joints and AI. The checklist encouraged full search of an image but also prompted the participant to consider and complete other aspects of the image interpretation which the AI would not have the capacity to consider. An example of the checklist sections is included in Figure 3. The checklist in full has been included as supplementary information.

### Questionnaire

The questionnaire was formed by the research team which included several AI researchers, radiographers and a consultant reporting radiographer. Radiographers and academia staff were consulted to pilot the questionnaire. Feedback was sought, and changes to wording and structure were made to reflect the feedback. An electronic questionnaire containing open and closed questions was completed by participants. This helped to gain an insight into the individual's experience/ field of practice and the participant's thoughts on the use of the checklist, including areas for improvement.

### Diagnosis and confidence level

Participants were asked to type the diagnosis and select a confidence level in their diagnosis immediately following their interpretation. Participants typed the diagnosis immediately below the presented examination. Diagnosis and confidence levels were calculated based on information provided from participants following their image interpretations. Participants were scored on each diagnosis provided. The figures given in Table 1 represent the percentage of cases which were correctly identified as normal or the pathology present. Participants were asked to provide a confidence level (between 1 and 10) on their diagnosis for each examination. This was then converted to a percentage for all examinations before and after checklist. These figures are presented in Table 1.

**Table 1:** Pre‐/post‐checklist diagnosis and the pre‐/post‐checklist confidence for each of the participants in this study.

Participant ID	Pre‐checklist correct diagnosis (%)	Post‐checklist correct diagnosis (%)	Pre‐checklist confidence (%)	Post‐checklist confidence (%)
100	80	80	82	90
101	80	80	78	86
111	80	80	32	42
112	80	80	66	64
116	80	80	72	58
131	80	40	90	74
202	60	40	68	66
303	80	80	86	86
404	80	80	74	88
505	40	40	50	50
606	80	80	54	72
707	60	60	70	84
808	60	40	30	14
909	80	80	82	80

Grey: Changed their mind post‐checklist and the correct diagnosis percentage decreased. Blue: Confidence in diagnosis increased from pre‐checklist to post‐checklist. Orange: Confidence in diagnosis decreased from pre‐checklist to post‐checklist.

### Data analysis

Descriptive statistics and tests for normality were performed. A series of Mann–Whitney U‐tests were performed once data was deemed not normally distributed. Thematic analysis was conducted on the textual responses according to the recommendations by Braun and Clarke.^26^

## Results

A total of 14 radiographers completed the study comprising five radiographic examinations. Participants had an average of 20 years of experience working clinically in a range of 3–40 years of clinical work experience. Table 1 depicts the pre‐/post‐checklist diagnosis and the pre‐/post‐checklist confidence for the participants of this study. The diagnosis is displayed as a percentage of the examinations for which the correct diagnosis was provided. Three participants changed their mind after using the checklist and their rate of correct diagnosis decreased from pre‐checklist to post‐checklist by 20–40% (highlighted in light grey). However, the majority of the participants did not appear to change their minds regarding their diagnosis post‐checklist. Five radiographers' confidence in diagnosis increased from pre‐checklist to post‐checklist by 12–14% (highlighted in light blue). Six radiographers had a decrease in their confidence from pre‐checklist to post‐checklist by 2–14% (highlighted in light orange), suggesting their confidence has been adversely affected by the checklist intervention when using AI assistance.

There were no significant differences found for correct diagnosis or confidence in diagnosis pre‐ and post‐use of a checklist (Table 2).

**Table 2:** Statistical testing of pre‐/post‐checklist diagnosis and the pre‐/post‐checklist confidence for each of the participants in this study.

Descriptive statistics and statistical testing ‐>	Skewness	Kurtosis	Normally distributed or not	Mann–Whitney test
Pre‐check list examination scores	−1.69	2.21	Not normally distributed	0.57
Post‐checklist examination scores	−0.86	−1.35	Not normally distributed	
Pre‐check list confidence level scores	−0.93	0.01	Not normally distributed	0.70
Post‐checklist confidence level scores	−1.33	1.78	Not normally distributed	

The participants expressed a broad range of views on the AI tool (Table 3). Nine of the participants found the AI section of the tool least useful. The participants felt that more information regarding AI diagnosis would be ideal as it would provide more clarity in AI feedback. Five participants found AI to be helpful as it helped to investigate the radiographic image interpretation more closely and consider their initial diagnosis. Some participants felt that AI was a work in progress and that it did not change their opinion regarding radiographic image interpretation.

**Table 3:** Participants' thoughts and perspectives of the artificial intelligence (AI) tool of the study.

Participant ID	Participants perspectives on the AI tool
100	*‘Unclear AI feedback’*.
101	*‘Couldn't trust AI’*.
111	*‘Image viewing conditions not ideal’*.
112	*‘Errors by AI diagnosis’*.
116	*‘Confused by AI diagnosis’*.
131	*‘More information regarding AI diagnosis should be shown’*.
202	*‘AI diagnosis was not helpful’*.
303	*‘AI diagnosis doesn't change my opinion’*.
404	*‘AI diagnosis helped me to look more carefully’*.
505	*‘AI diagnosis helped me to pay more attention to the details’*.
606	*‘Did not rely on AI diagnosis’*.
707	*‘AI is still a work in progress’*.
808	*‘AI diagnosis should be more specific’*.
909	*‘AI diagnosis made me rethink my diagnosis of the image’*.

### Feedback received

The checklist was deemed to have varying levels of usefulness. Fifty per cent of participants found the bony detail and joints section to be the most useful in radiographic image interpretation (*n* = 7), followed by the soft tissues section (35.7%) and lastly the artificial intelligence (AI) section (7.1%) and alignment section (7.1%). Seven participants selected the artefacts and normal variants section as the least useful for radiographic image interpretation. Participants varied in their opinions on the influence of AI on the image interpretation process. Eleven out of fourteen participants did not feel the AI influenced their image interpretation process. Participants' views of the aspects of the checklist that could be improved were collated. Eleven participants reported the AI section could benefit most from improvements, followed by three participants reporting that the cartilage and joints section of the check list could be improved. Only one participant felt the alignment section and the soft tissues section required improvements. Nine participants found the checklist most useful and five participants found the AI element to be most useful.

## Discussion

Overall, the performance of radiographers completing radiographic image interpretation was affected by the checklist. Participants' certainty in diagnosis decreased by 20–40%. There were only three participants who changed their diagnosis following the check list. The current checklist caused participants to question the initial diagnosis they provided; however, a checklist tailored to meet the users' needs may help to avoid errors and biases in using AI and, in doing so, improve patient management and diagnosis. With the use of a checklist in image interpretation, the radiographer completing PCE may enhance their skills and avoid bias, to develop a recognised role within the healthcare team. As AI is a rapidly progressing and important topic in radiographic image interpretation, a checklist aiding its use in determining the diagnosis certainty may be more beneficial. Keeping in mind conclusions drawn by Savadjiev et al.,^27^ it is crucial that AI based methods and tools are validated to ensure that clinical outcome measures remain the priority in the measurement of success. Therefore, validation of a checklist or tool, to be used with AI, would be required before implementation in clinical practice to maximise its use and effect.

Moreover, there was an increase in confidence noted for some participants, but a decrease in confidence after checklist intervention for other participants. As the checklist did not improve the overall confidence in radiographers significantly, more AI feedback and greater information supplied to researchers may help build an enhanced and robust AI model and this in turn may allow radiographers' confidence to increase in both their interpretation and in using AI in clinical practice, ultimately improving the workflow and accuracy of interpretation. The AI and alignment sections were found beneficial by few participants, indicating this as an area for improvement in the development of the checklist. Participants also indicated their preference for more direction and detail in the AI assistance provided. This concurs with the findings of Rainey et al.^28^ where it is stated trust levels could be improved with explainable AI solutions.

The use of checklists has been proven useful in image interpretation tasks.^29^ The potential impact of the checklist is that it will provide assistance and reassurance to those using AI guidance in image interpretation, allowing the users to further trust the information provided to them and their own judgement on diagnosis. The use of the checklist may encourage autonomy and confidence following further research and training in the use of AI, therefore making these findings highly relevant to all radiographers involved in the image interpretation tasks. The findings of this study were in slight disagreement with the brief on ‘Evidence on Use of Clinical Reasoning Checklists for Diagnostic Error Reduction’.^15^ Some participants changed their mind to the wrong diagnosis, suggesting the checklist caused doubt in some participants. Checklists may be useful, as some participants were more confident following the intervention; however, it caused others to change their mind to an incorrect diagnosis, suggesting they may not be useful for all clinicians. There is potential for the checklist and study findings to be used in educational interventions for those using AI in image interpretation. Radiographers are encouraged to participate in AI training programs and thus maximise the use of AI in the profession and image interpretation. However, care must be taken to avoid becoming heavily reliant on the information provided or being reluctant to use it. The novel checklist that has been developed in this study for the use in image interpretation when using AI can be enhanced and the findings in this study can be used in clinical practice.

Only three participants felt the AI feedback influenced their image interpretation process, and 11 participants stated that the AI section of the checklist could be improved. This suggests the AI section of the checklist could be used more efficiently to allow the user to integrate it more effectively in informing their image interpretation process. The majority of participants found the checklist most useful in their image interpretation, possibly indicating its use with a different approach to the AI section would allow the checklist to be more favourable in image interpretation assistance. The image viewing conditions were cited as a limitation of the study despite efforts made to improve the image viewing conditions. A further limitation was only five examinations were presented to participants, and this is a small sample to quantify performance or accuracy.

## Conclusion

A novel checklist has been developed for use in image interpretation and to aid the interpretation of images when using AI. The checklist for use with AI in MSK image interpretation contained elements, which were useful to the user but further developments can be made to meet the specific needs of the image interpreter. Changes will be made to facilitate the use of the checklist and to enhance its use in clinical practice. Conflicting results were found, and some participants improved in confidence with the checklist, whereas others did not. The checklist appeared to cause some participants to question their decision and to change their mind to an incorrect diagnosis. This suggests a checklist may be useful to some clinicians but not to all. Further research will focus on the iterative development of the checklist to incorporate the beneficial aspects found in this study and modifications and additional items based on the end‐user's preferences. Further investigations using a larger number of examinations and interpretations may help validate the findings and determine clinical utility. Future research could perhaps help to determine the characteristics of those who wouldor would not benefit from the use of a checklist.

### Ethics

Ethical permission for this study has been granted by Ulster University Nursing and Health Research Ethics Filter Committee FCNUR‐20‐035. Ethical permission for the use of the clinical dataset images has been granted previously to use the images for research purposes (Monash University, Clayton, Australia, 2011).

## Funding Information

The College of Radiographers Research Industry Partnership Award Scheme (CoRIPS) provided funding for this study.

## Conflict of Interest

No conflicts of interest.

## Supporting information


**Data S1.** Image Interpretation Checklist.

## Data Availability

The data that support the findings of this study are available from the corresponding author upon reasonable request.
