# Supplementary material for: An evaluation of a checklist in musculoskeletal radiographic image interpretation when using artificial intelligence
Source: J Med Radiat Sci. 2024 Dec 20;72(2):209–16. doi: 10.1002/jmrs.850 (PMC13014785; doi:10.1002/jmrs.850)
Supplement: Supplementary file 1 — Data S1. Image Interpretation Checklist. [file JMRS-72-209-s001.docx]

**Title of the study:**

**An evaluation of a checklist in Musculoskeletal (MSK) radiographic image interpretation when using Artificial Intelligence (AI)**

**Image Interpretation Checklist**

**Alignment**

**Check each joint present on the image**

**Is there misalignment associated with any of the joints? YES**

**NO**

**If yes, search the bony areas surrounding the joint area**

**Is there any subsequent injury to the bone adjacent to the affected joint YES**

**NO**

**Bony detail and joints**

**Is the cortex of each bone NORMAL**

**ABNORMAL**

**Search areas beyond those highlighted by the AI**

**Is there an abnormality in the cortex bone beyond these? YES NO**

**Is there an abnormality in the bony trabeculae beyond these? YES**

**NO**

**Are there any abnormalities present? YES**

**NO**

**Cartilage and joints**

**Check each joint present on the image**

**Is there any abnormality surrounding the joint area? YES**

**NO**

**Soft tissues**

**Look around the soft tissue border, beyond those highlighted by the AI**

**Is it? NORMAL**

**ABNORMAL**

**Look within the soft tissue structures:**

**Is there evidence of attenuation differences? YES**

**NO**

**Is there evidence of soft tissue damage? YES**

**NO**

**Is there evidence of effusion? YES**

**NO**

**Is there evidence of calcification? YES**

**NO**

**Are there any abnormalities present? YES**

**NO**

**Artefacts and normal variants**

**Is there an artefact present on the image? YES**

**NO**

**If yes, is this artefact obscuring anatomy/pathology? YES**

**NO**

**Is there a normal variant on the image? YES**

**NO**

**Artificial Intelligence**

**Have you reviewed the area of interest identified by the AI? YES**

**NO**

**Do you agree with the localisation of the heatmap? YES**

**NO**

**NEGATIVE**

**POSITIVE**

**Do you agree/disagree with the AI decision? AGREE**

**DISAGREE**

**PARTLY**

**Please comment**

**Are you confident bony integrity is intact in the area identified by the AI? YES**

**NO**

**Please comment where necessary**

**Is the bony integrity intact in all areas beyond that identified by the AI? YES**

**NO**

**Please comment where necessary**
